# High throughput sequencing revealed enhanced cell cycle signaling in SLE patients

**DOI:** 10.1038/s41598-022-27310-8

**Published:** 2023-01-04

**Authors:** Mingyue Yang, Peisong Wang, Tao Liu, Xiaojuan Zou, Ying Xia, Chenxu Li, Xiaosong Wang

**Affiliations:** 1 Laboratory for Tumor Immunology, Translational Medicine Department, First Hospital of Jilin University, Changchun, 130021 China; 2 Thyroid Surgery Department, General Surgery Center, First Hospital of Jilin University, Changchun, 130021 China; 3 Department of Rheumatology and Immunology, First Hospital of Jilin University, Changchun, 130021 China; 4 Present Address: Institute of Translational Medicine, First Hospital of Jilin University, No.519 Dongminzhu Street, Changchun, 130021 China

**Keywords:** Autoimmunity, Cytokines, Rheumatology, Systemic lupus erythematosus

## Abstract

The multi-system involvement and high heterogeneity of systemic lupus erythematosus (SLE) pose great challenges to its diagnosis and treatment. The purpose of the current study is to identify genes and pathways involved in the pathogenesis of SLE. High throughput sequencing was performed on the PBMCs from SLE patients. We conducted differential gene analysis, gene ontology (GO) analysis, kyoto encyclopedia of genes and genomes (KEGG) analysis, and quantitative real-time PCR (qRT-PCR) verification. Protein–protein interaction (PPI) analysis, alternative splicing analysis, and disease correlation analysis were conducted on some key pathogenic genes as well. Furthermore, si-*CDC6* was used for transfection and cell proliferation was monitored using a cell counting kit-8 (CCK-8) assay. We identified 2495 differential genes (1494 upregulated and 1001 downregulated) in SLE patients compared with healthy controls. The significantly upregulated genes were enriched in the biological process-related GO terms of the cell cycle, response to stress, and chromosome organization. KEGG enrichment analysis revealed 7 significantly upregulated pathways including SLE, alcoholism, viral carcinogenesis, cell cycle, proteasome, malaria, and transcriptional misregulation in cancer. We successfully verified some differential genes on the SLE pathway and the cell cycle pathway. *CDC6,* a key gene in the cell cycle pathway, had remarkably higher MXE alternative splicing events in SLE patients than that in controls, which may explain its significant upregulation in SLE patients. We found that *CDC6* participates in the pathogenesis of many proliferation-related diseases and its levels are positively correlated with the severity of SLE. Knockdown of *CDC6* suppressed the proliferation of Hela cells and PBMCs from SLE patients in vitro. We identified SLE-related genes and their alternative splicing events. The cell cycle pathway and the cell cycle-related biological processes are over-activated in SLE patients. We revealed a higher incidence of MXE events of *CDC6*, which may lead to its high expression in SLE patients. Upregulated cell cycle signaling and *CDC6* may be related to the hyperproliferation and pathogenesis of SLE.

## Introduction

Systematic lupus erythematosus (SLE) is a complex autoimmune disease involving multiple organs and systems^1^. Extensive organ damage may happen in the early stage of SLE, which can seriously affect a patient’s quality of life^2–5^. The main features of SLE include the production of autoantibodies and the deposition of immune complexes^6^. Immune complexes may be deposited in the skin, joints, renal tubule, glomeruli, and other parts of the patients^7^. Comprehensive autoAb screening revealed that the antigens targeted by the autoAbs were most significantly enriched in cell death, cell cycle, and DNA repair pathways^8^. Therefore, SLE may also be a cell proliferation disease^9^. We speculate that the hyperactivation of cell cycle pathway may lead to B cell proliferation and massive production of autoantibodies. The in-depth study of genes and pathways involved in the pathogenesis of SLE will lay a foundation for the effective diagnosis and treatment of the disease.

Peripheral blood mononuclear cells (PBMCs) of patients have been widely used to study the pathogenesis of systemic immune diseases such as SLE and rheumatoid arthritis (RA)^10–13^. High throughput transcriptome sequencing technology with perfect quantitative function was applied to study gene expression in SLE^14–16^. Therefore, we used this method to study the PBMCs from SLE patients. The differential genes were analyzed by gene ontology (GO) enrichment analysis, kyoto encyclopedia of genes and genomes (KEGG) analysis. We further studied the protein–protein interaction (PPI), alternative splicing, etc*.* to explore the key pathogenic genes and pathways of SLE.

## Methods

### Sample collection and clinical information

We recruited 78 SLE patients and 50 matched healthy controls for the current study. All of the patients met at least 4 of the 11 diagnostic criteria for SLE that were revised by the american college of rheumatology (ACR)^17^. Clinical data of newly diagnosed SLE patients from August 2018 to November 2022 were collected at the First Hospital of Jilin University (Additional File 1–2: Table S1, S2). 20 SLE patients and 10 healthy controls were randomly selected for mRNA sequencing. Samples from 58 SLE patients and 40 healthy controls were used for validation and function assay. All of the patients and healthy controls were Asian, and they had no alcoholism, viral carcinogenesis, proteasome, malaria, cancer, and other diseases at the time of sample collection. PBMCs were isolated from whole blood by density gradient centrifugation (Lymphopre, Axis-Shield, Scotland)^18^. TRIzol reagent (1 ml; Invitrogen, Carlsbad, CA, USA) was added to 5 × 10^6^ PBMCs and stored at − 80 °C. The immunoturbidimetric kits (Zhongyuan Biotech Holdings, China) were used to detect the serum levels of IgM, IgG, and IgA for the SLE patients.

### Ethics approval and consent to participate

Ethical approval for the current study was obtained from the Institutional Medical Ethics Review Board of the First Hospital of Jilin University (reference number: 2017–087). Informed consent for enrolment was obtained from all selected participants or their guardians. All procedures complied with the Declaration of Helsinki.

### Sequencing

Total RNA was extracted using TRIzol (Invitrogen, Carlsbad, California, USA) according to the manufacturer’s instructions. RNA degradation and contamination were monitored on 1% agarose gels. We tested RNA purity using NanoPhotometer^®^ (IMPLEN, CA, USA) and measured RNA concentration using Qubit^®^ RNA Assay Kit on a Qubit^®^ 2.0 Fluorometer (Life Technologies, CA, USA). RNA integrity was assessed using RNA Nano 6000 Assay Kit on a Bioanalyzer 2100 system (Agilent Technologies, CA, USA)^19,20^.

We used 3 μg RNA per sample as input material for sample preparation. Firstly, ribosomal RNA was removed by Epicentre Ribo-zero™ rRNA Removal Kit (Epicentre, USA) and rRNA-free residues were cleaned up by ethanol precipitation. Subsequently, sequencing libraries were generated using NEBNext^®^ Ultra™ Directional RNA Library Prep Kit (NEB, USA) following the manufacturer’s recommendations, and index codes were added to attribute sequences to each sample. The clustering of the index-coded samples was performed on a cBot Cluster Generation System using TruSeq PE Cluster Kit v3-cBot-HS (Illumia) according to the manufacturer’s instructions. We assessed the quality of sequencing library on Agilent Bioanalyzer 2100 system. Finally, sequencing was performed with Illumina HiSeq X Ten (paired-end 150-bp reads).

### Sequencing data analysis

We firstly processed raw data (raw reads) of fastq format through in-house perl scripts. In this step, we obtained clean data(clean reads) by removing reads containing adapter, reads containing ploy-N and low quality reads from raw data. At the same time, we calculated Q20, Q30 and GC content of the clean data. All the downstream analyses were based on the clean data with high quality.

Ballgown suite was used for interactive exploration of the transcriptome assembly, visualization of transcript structures and feature-specific abundances for each locus, and post-hoc annotation of assembled features to annotated features^21^. Reference genome and gene model annotation files were downloaded from the Ensembl database (Homo_sapiens.GRCh38.94). We built the index of the reference genome using HISAT2 v2.0.4 and aligned paired-end clean reads to the reference genome using HISAT2 v2.0.4^22^. HISAT2 was run with ‘–rna-strandness RF’, other parameters were set as default. We assembled the mapped reads of each sample by StringTie (v1.3.3)^23^ in a reference-based approach. The software used for difference analysis is edgeR (3.0.8)^24^.Transcripts with a corrected-*P* < 0.05 were assigned as differentially expressed.

GO enrichment analysis of differentially expressed genes was implemented by the GOseq R package, in which gene length bias was corrected^25^. GO terms with corrected-*P* < 0.05 were considered significantly enriched. KEGG is a database resource for understanding high-level functions and utilities of the biological system^26^, from molecular-level information, especially large-scale molecular datasets generated by genome sequencing and other high-throughput experimental technologies (http://www.genome.jp/kegg/). We used KOBAS software to test the statistical enrichment of differential expression genes in KEGG pathways^19,20,27^.

We used rMATS (v3.2.1) to detect the differential alternative splicing events in replicate RNA-Seq data^28^. Alternative splicing events were classified to five basic types by software Asprofile v1.0. The expression values for all transcripts located in the same gene locus were added together to represent the expression level of that particular gene. Then, using the gene expression matrix prepared, we used an online transcriptome-based immune cell number prediction tool ABIS (https://giannimonaco.shinyapps.io/ABIS/) to estimate the proportion of different immune cell types.

### Quantitative real-time PCR (qRT-PCR)

We treated cells with TRIzol reagent (Invitrogen, Carlsbad, California, USA). RNA was extracted using TransZol Up Plus RNA Kit (TransGen Biotech, Beijing, China). We used Trans Script All-in-One First-strand cDNA Synthesis Supermix for qPCR Kit (TransGen Biotech, Beijing, China) in RNA reverse transcription. qRT-PCR was then performed as previously described^18,29^.

### Cell transfection

Small interference RNA against *CDC6* (si-*CDC6*) and its negative control (NC) were assembled by IBSBIO (Shanghai, China). Hela cells were seeded in 12-well plate (5 × 10^5^ cells/well/ml for qRT-PCR assay) or 96-well plates (5 × 10^3^ cells/well/100μL for proliferation assay) in Dulbecco's modified eagle medium (Corning, NY, USA) with 10% fetal bovine serum (BIOIND, Kibbutz Beit Haemek, Israel). PBMCs were plated in 12-well plate (5 × 10^5^ cells/well/ml for qRT-PCR assay) or 96-well plates (5 × 10^3^ cells/well/100μL for proliferation assay) in RPMI1640 (Corning, NY, USA) with 10% patients’ serum. After plating and overnight culture, all oligonucleotides were transfected using Lipofectamine 3000 (Invitrogen, CA, USA), and non-transfected cells were used as the control. 48 h later, cells were used for qRT-PCR or proliferation assay.

### Cell counting kit-8 (CCK-8) assay

To detect cell proliferation, 48 h after transfection in 96-well plates, 10 μL CCK-8 reagent (MedChemExpress, Shanghai, China) was added into each well and incubated at 37 °C for 2 h. Finally, the absorbance at 450 nm was detected with the Synergy H1 Hybrid Reader (Biotek, Winooski, VT, USA).

### Statistical methods

GraphPad Prism 8.0 was used for statistical analysis and picture drawing (GraphPad Software, San Diego, USA). In order to compare different matrices, we used the expression level of each gene to generate an unsupervised heat map. Wilcoxon signed-rank test was used to compare paired samples, and Mann–Whitney *U* test was used to compare unpaired samples. Nonparametric Spearman rank correlation test was applied to determine the correlations. A *P* value below 0.05 was considered significant.

Protein–protein interaction (PPI) patterns were analyzed using STRING database (http://string-db.org/; version 10.5) with a confidence score > 0.9. A gene-related disease enrichment map was generated on Open Targets Platform^30^ (https://www.targetvalidation.org, search keyword: *CDC6*) on September 6, 2021. The expressional distribution data of *CDC6* in the PBMCs of healthy people were downloaded from the Single Cell Portal website(https://singlecell.broadinstitute.org/single_cell/study/SCP345/ica-blood-mononuclear-cells-2-donors-2-sites, search keyword: *CDC6*) on September 6, 2021.

## Results

### Identification and classification of differentially expressed genes between the SLE group and control group

**Figure 1:**
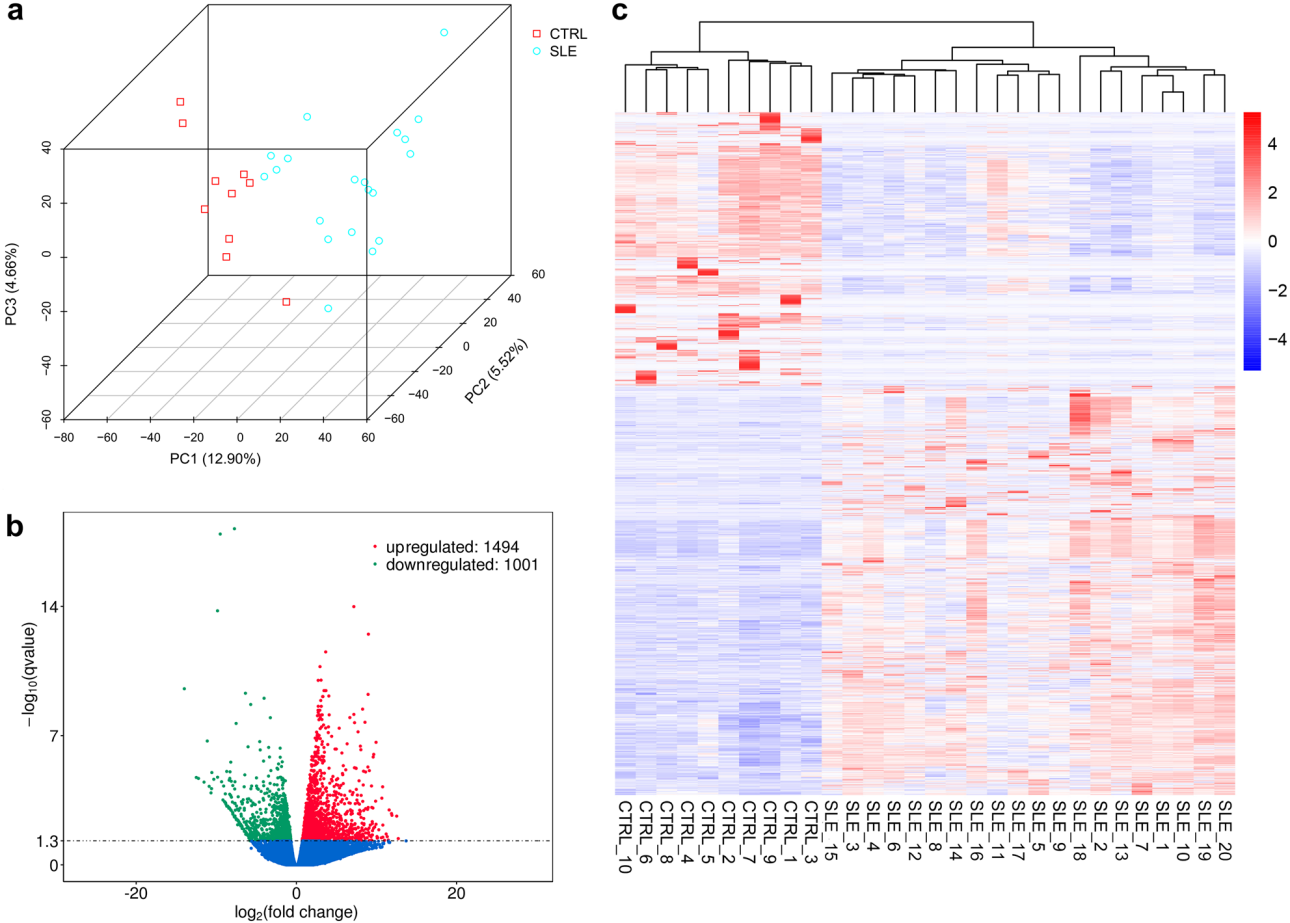
Sequencing samples and differentially expressed genes. **(a)** Principal component analysis (PCA) plot of the sequencing samples. 3D PCA is conducted to evaluate the clustering natures and repeatability of the samples. Percentages are the contribution ratios. **(b)** Volcano plot of the genes differentially expressed between SLE group and CTRL group. Each point represents one gene that is detectable in both groups. **(c)** The cluster of 2495 genes differentially expressed between the SLE group and the CTRL group. Corrected-*P* < 0.05.

Clinical information of the sequencing subjects was shown in Additional File 1: Table S1. There were no significant differences in gender or age between the two groups. We carried out principal component analysis (PCA) to assess the clustering nature of these samples. Data showed good repeatability and correlations (Fig. 1a). We identified 2495 differentially expressed genes (1494 upregulated genes and 1001 downregulated genes) between the SLE group and the control group (Fig. 1b). The clustering analysis of these genes was shown in Fig. 1c. The top 20 upregulated genes were *TTK*, *ETV7*, *NCAPG*, *TOP2A*, *ASPM*, *IFI44*, *DLGAP5*, *HIST1H3J*, *HIST1H3G*, *HESX1*, *PBK*, *USP18*, *IFI44L*, *HIST1H3C*, *HIST1H2AH*, *HIST2H4A*, *HIST1H3B*, *IFI27*, *HIST1H2BH* and *HIST1H1B* (Additional File 3: Table S3). The top 20 downregulated genes were *SPON2*, *TMEM8B*, *AUTS2*, *FYN*, *ADA2*, *TBC1D22A*, *AUTS2*, *ARHGAP45*, *MSANTD2*, *SCRN1*, *HSP90AB1*, *AGAP*1, *CAST*, *COL6A2*, *ACIN1*, *LARS2*, *MT-ND1*, *SARS2*, *ZC3HAV1* and *ZMYM3* (Additional File 4: Table S4).

**Figure 2:**
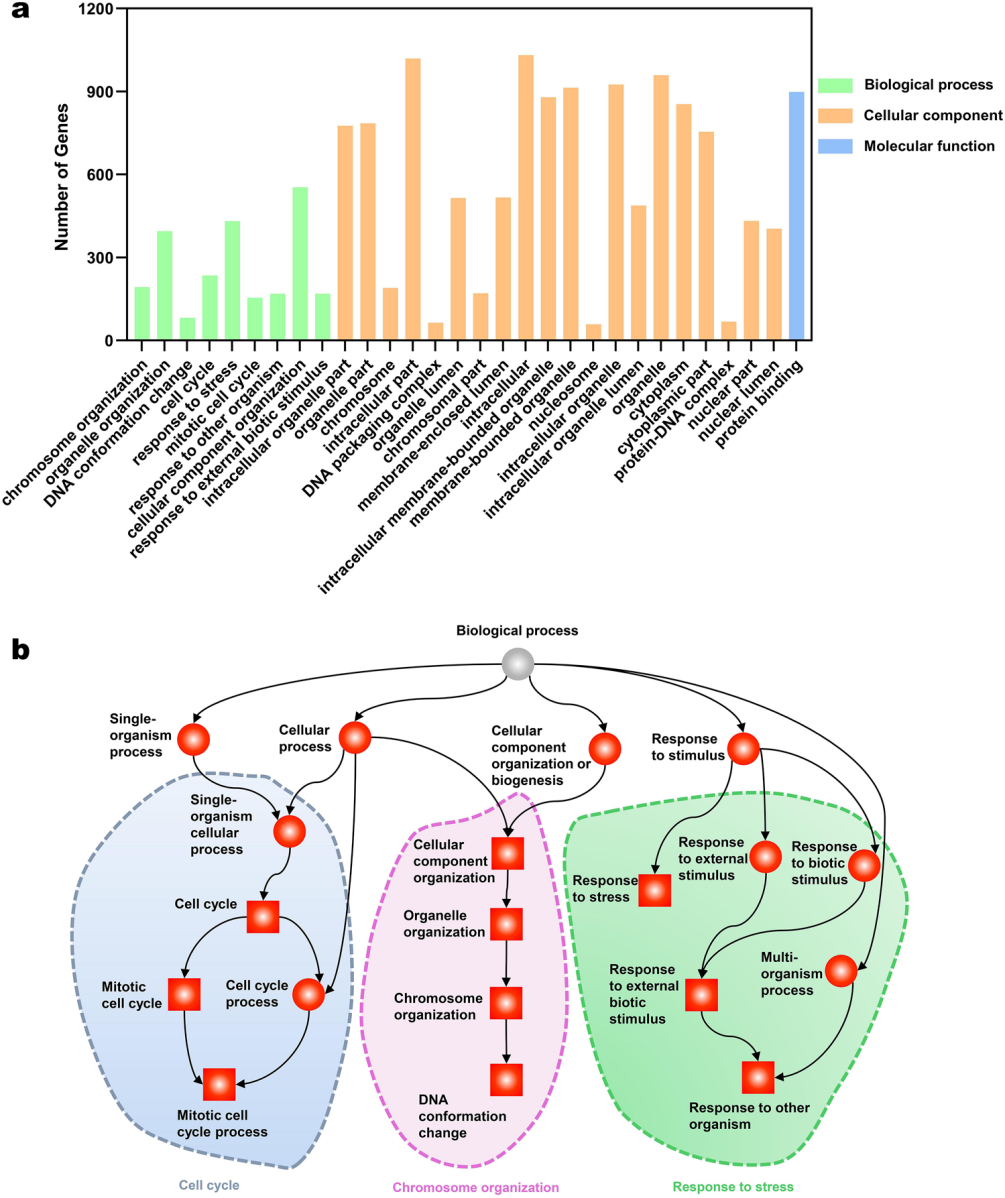
Gene Ontology (GO) analysis of the upregulated genes between systemic lupus erythematosus (SLE) group and control (CTRL) group. **(a)** Top 30 GO terms enriched in biochemical processes, cellular components, or molecular function. The numbers of differentially expressed genes between the SLE group and the CTRL group in each category were compared. **(b)** Directed acyclic graph of the upregulated GO terms in the biochemical processes class. Squares represent the top 10 GO terms based on corrected-*P* values; red squares or red circles represent terms with corrected-*P* < 0.05.

To explore the biological functions of these upregulated genes, we performed GO analysis (Fig. 2a). The top 30 upregulated GO terms were classified into biological process (BP) class, cellular component (CC) class, and molecular function (MF) class. Detailed information on each GO term was listed in Additional File 5: Table S5. The 20 GO terms of CC class are mainly enriched in DNA, chromosome, and nuclear-related terms. Furthermore, the terms of BP class were depicted as directed acyclic graphs in Fig. 2b to show their relationships. The significantly upregulated genes were highly enriched into 3 groups of GO terms including cell cycle, chromosome organization, and response to stress. The top 30 downregulated GO terms were classified into BP class, CC class, and MF class. Detailed information on each GO term was listed in Additional File 6: Table S6.

### KEGG pathway enrichment analysis

**Figure 3:**
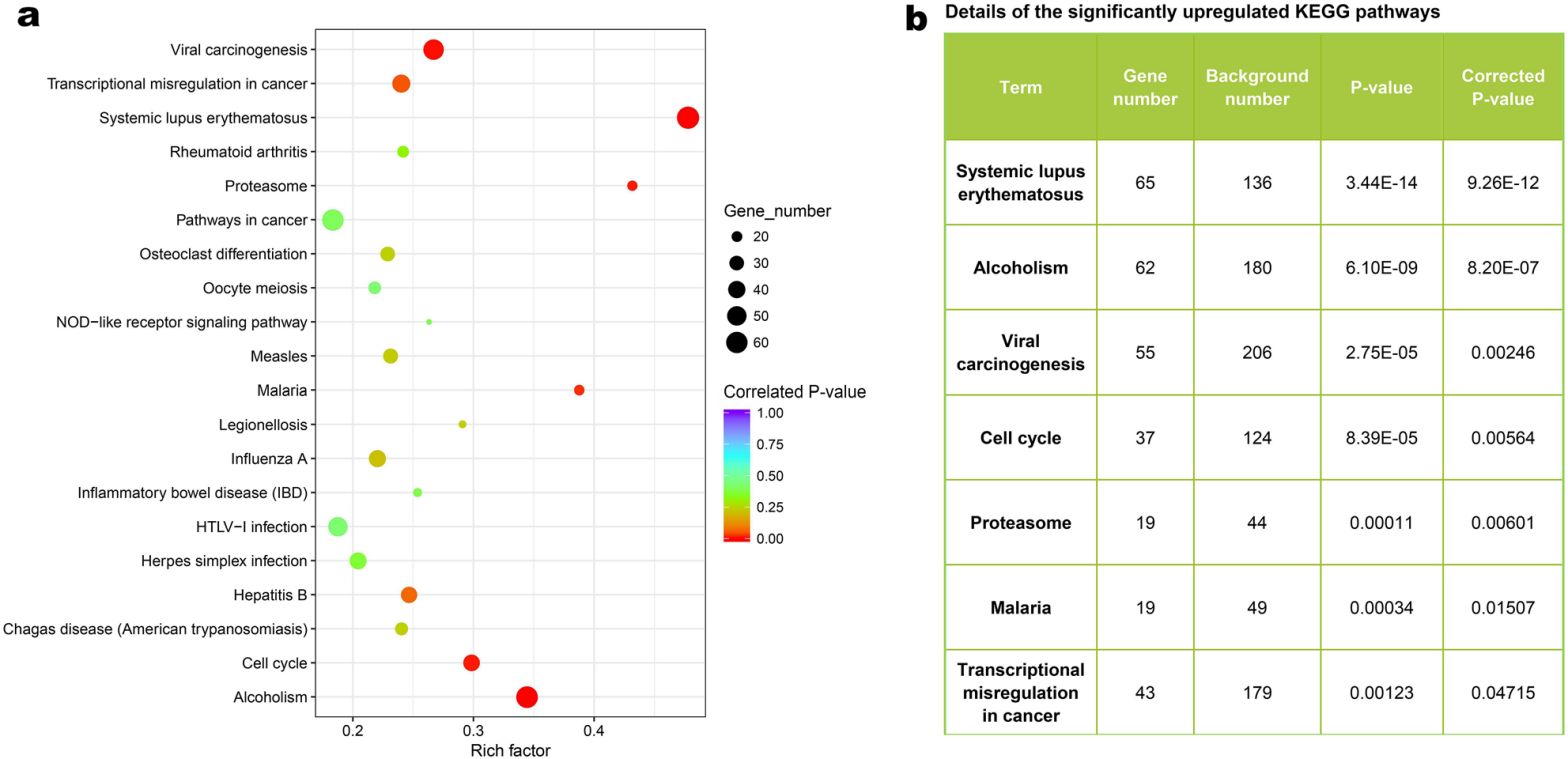
Kyoto encyclopedia of genes and genomes (KEGG) enrichment analysis. **(a)** Dot plot of pathway enrichment. The size of the dot represents the number of genes enriched in the pathway. The color of the dot represents the significance of the difference. **(b)** List of the significantly upregulated pathways in the systemic lupus erythematosus (SLE) group compared with the control (CTRL) group (corrected-*P* < 0.05).

The top 20 upregulated KEGG pathways were shown in Fig. 3a. Detailed information on seven significantly upregulated KEGG pathways can be found in Fig. 3b. The SLE pathway and cell cycle pathway were significantly upregulated (Fig. 3, corrected-*P* < 0.05). Top 20 downregulated KEGG pathways for SLE patients can be found in Additional File 7: Table S7.

**Figure 4:**
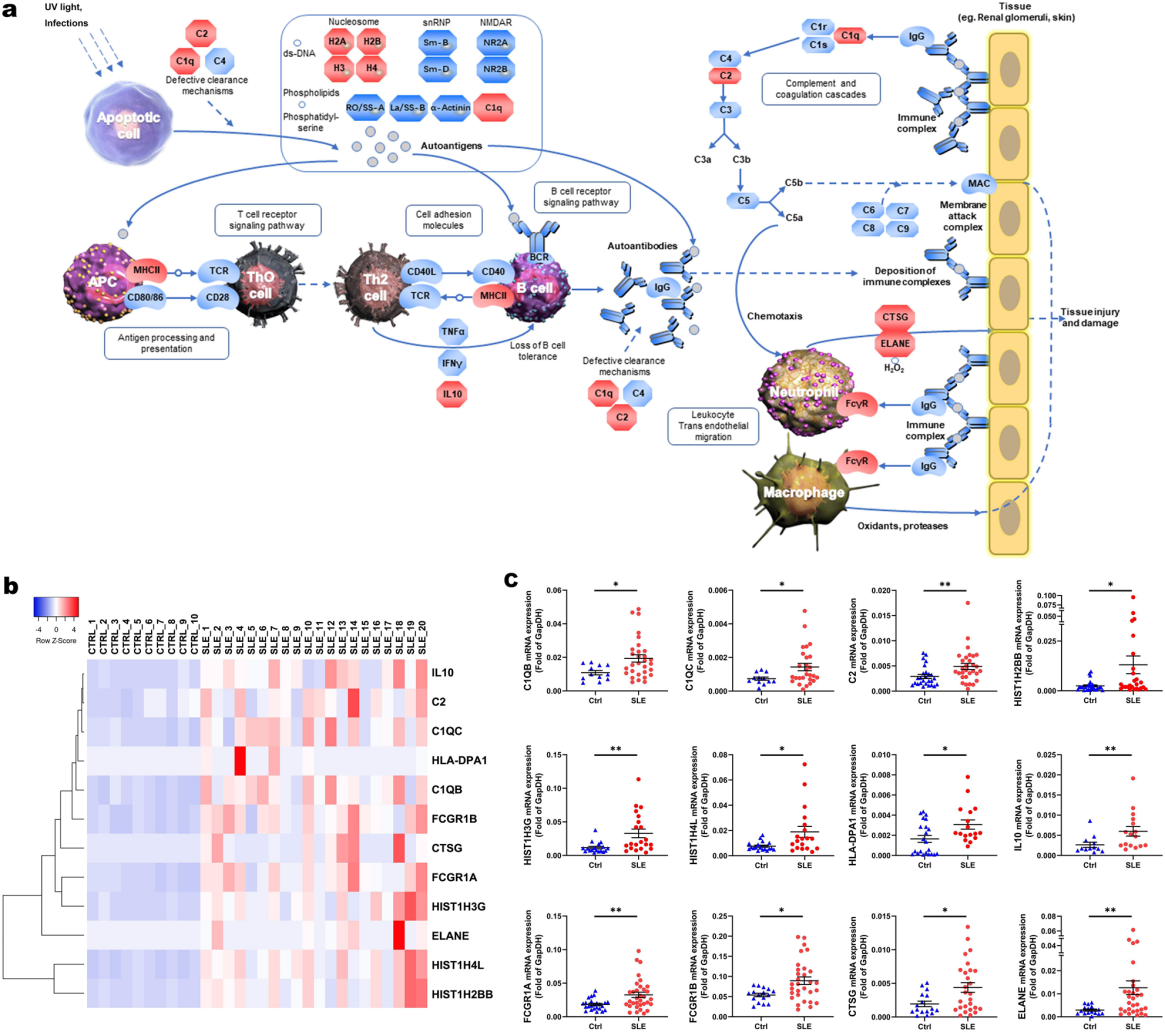
Upregulated genes mapped to systemic lupus erythematosus pathway. **(a)** Model diagram of systemic lupus erythematosus pathway, genes marked in red are significantly upregulated genes. **(b)** Cluster analysis was performed on 12 genes with significant differences between the systemic lupus erythematosus (SLE) group and the control (CTRL) group. **(c)** Quantitative real-time PCR (qRT-PCR) validation of the upregulated genes mapped to the SLE pathway. ***P < 0.001, **P < 0.01, *P < 0.05.

The SLE pathway is a pathway with an ID number of map 05322 (https://www.kegg.jp/entry/map05322, Additional File 8: Figure S1). Sixty-four upregulated genes (Additional File 9: Table S8) were identified in the SLE pathway (Fig. 4a). These genes are involved in autoantigens formation, lymphocyte activation, immune complex formation and deposition, and neutrophils/macrophages migration. Histone translation regulates DNA accessibility, which is related to the development of diseases.

Complement and coagulation cascades pathway are upregulated in SLE. *C1QA*, *C1QB*, *C1QC*, and *C2* were significantly upregulated. Figure 4b showed the cluster analysis on the significantly upregulated genes of *C1QB*, *C1QC*, *C2*, *HIST1A2BB*, *HIST1H3G, HIST1H4L*, *HLA-DPA1*, *IL10*, *FCGR1A*, *FCGR1B*, *CTSG*, and *ELANE.* Validation experiments confirmed that these genes were upregulated in the PBMCs of SLE patients (Fig. 4c).

**Figure 5:**
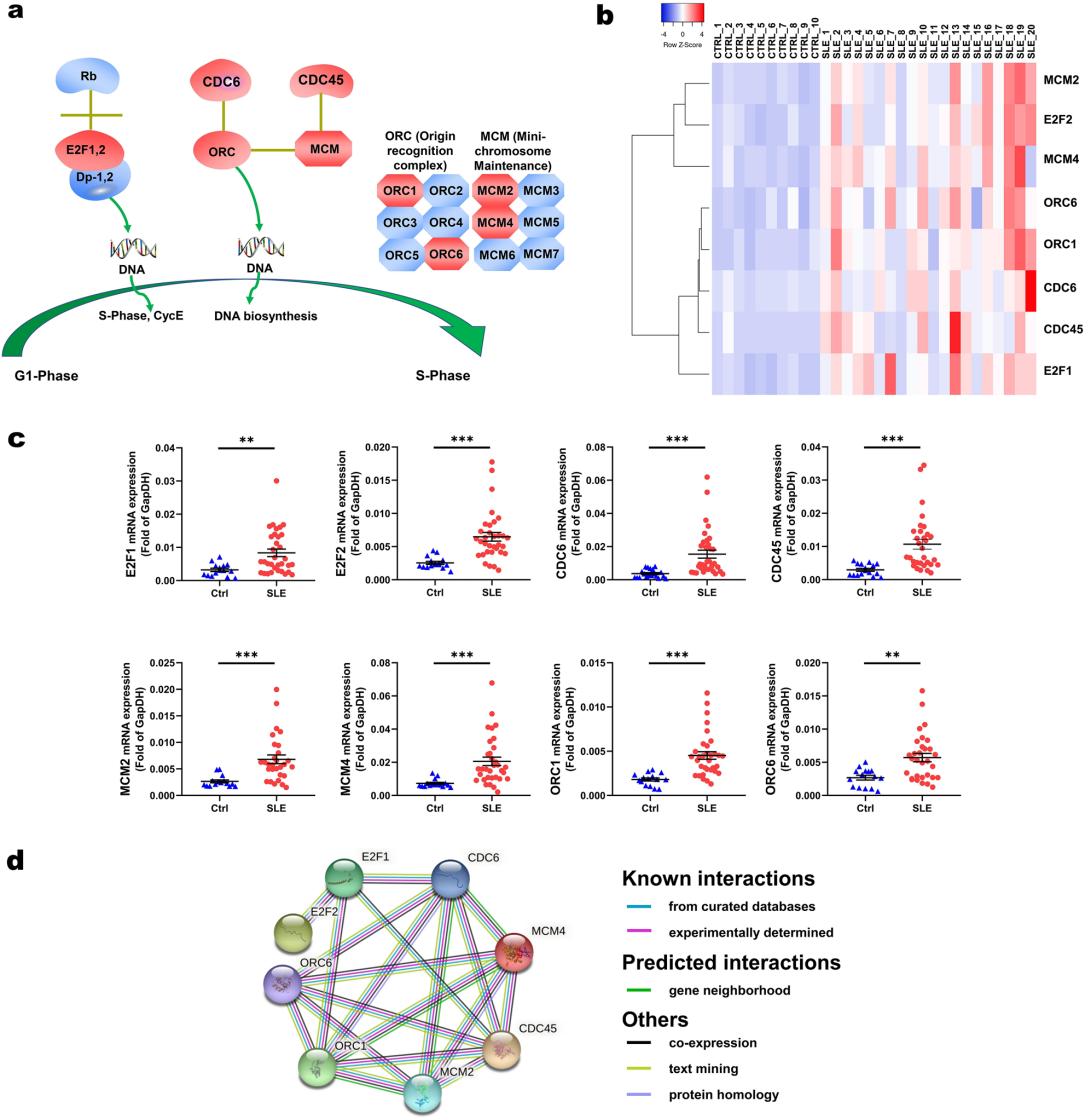
Upregulated genes mapped to cell cycle pathway. **(a)** Model diagram of a part of the cell cycle pathway, genes marked in red are significantly upregulated genes. **(b)** Cluster analysis was performed on 8 genes with significant differences between the systemic lupus erythematosus (SLE) group and the control (CTRL) group. **(c)** Quantitative real-time PCR (qRT-PCR) validation of some differentially expressed genes mapped to cell cycle pathway. ***P < 0.001, **P < 0.01, *P < 0.05. **(d)** Diagram of protein–protein interaction (PPI) network showing the evidence of the relationships among the proteins. The known or predicted 3D structure of the protein was shown inside the circles. The types of interaction evidence are labeled.

Genes (*RBX1*, *E2F3*, *CHEK1*, *BUB1*, etc., Additional File 8: Table S8 ) on the cell cycle pathway were upregulated in SLE patients. Among them, the members of the *E2F* family (*E2F1*, *E2F2*), *CDC* family (*CDC6*, *CDC45*), *MCM* family (*MCM2*, *MCM4*), and *ORC* family (*ORC1*, *ORC6*) participate in DNA biosynthesis (Fig. 5a, Additional File 10: Figure S2). Figure 5b showed the cluster analysis on them. qRT-PCR validated that *E2F1*, *E2F2*, *CDC6*, *CDC45, MCM2*, *MCM4*, *ORC1*, and *ORC6* were significantly upregulated in SLE patients (Fig. 5c). We mapped the interactions and evidence among these proteins as a protein–protein interaction (PPI) network (Fig. 5d).

### Alternative splicing events between the SLE group and control group

**Figure 6:**
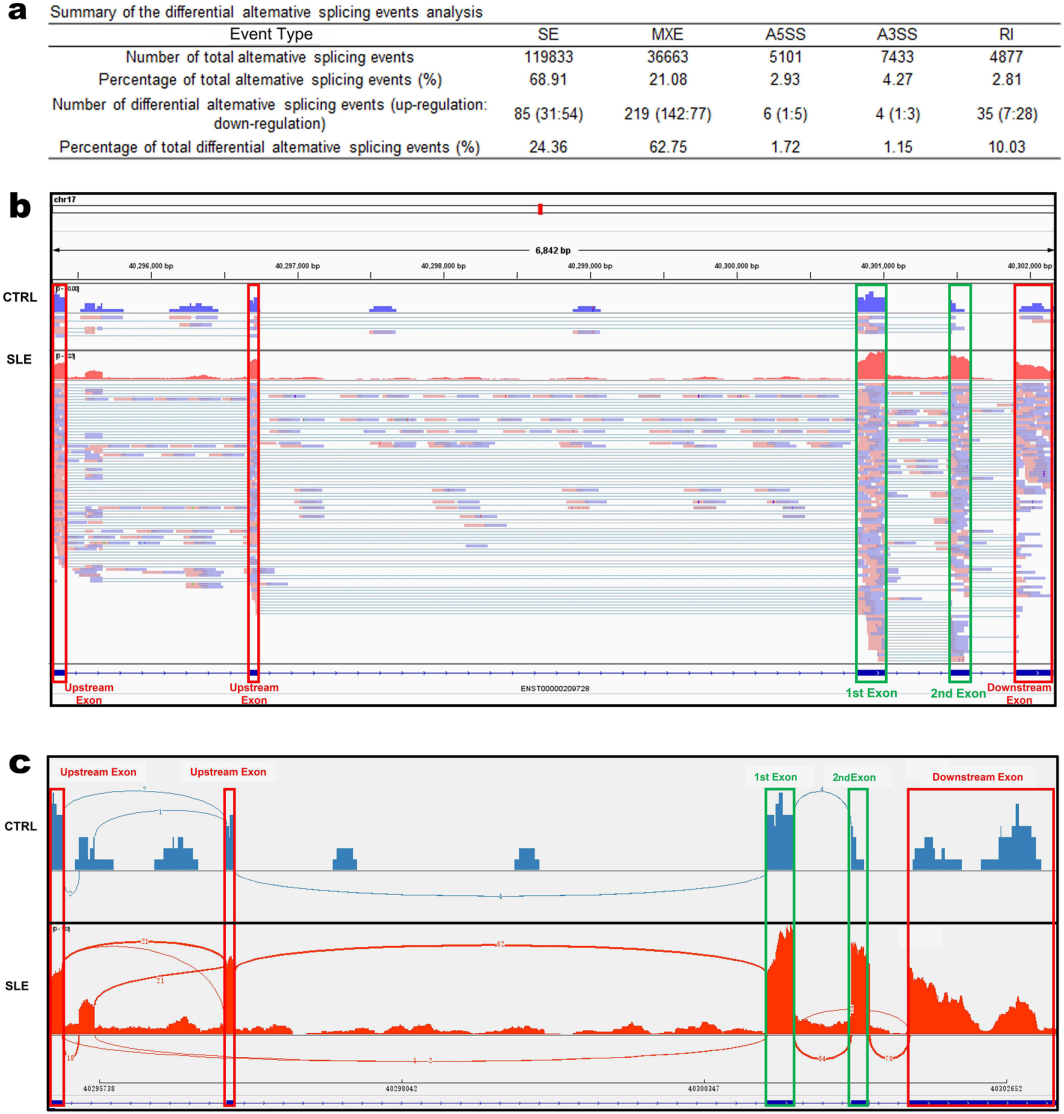
Differential alternative splicing events. **(a)** Summary of the differential alternative splicing event analysis results. **(b)** Read distribution plot of cell division cycle 6 (*CDC6*) with differential isoform expressions due to mutual exclusive exon (MXE). The histograms represent the abundance of the reads (blue represents CTRL1, red represents SLE19). Reads track, which directly displays the comparison of reads, is shown below the histograms. The green boxes represent the 1st exon or the 2nd exon where MXE occurs. **(c)** Sashimi plot is taken from the IGV Viewer that shows an MXE of the gene *CDC6* that occurred in CTRL1 and SLE19. All of the splice junctions detected were labeled, and the numbers on the arc represent the read depths of the junctions.

More than 90 percent of human genes have variable splicing, which plays an important role in the transmission of life information^31^. Compared with the control group, MATS analysis revealed 349 differential alternative splicing events in the SLE group (Fig. 6a) and 219 of them (62.75%) belonged to mutual exclusive exon (MXE). Among these genes with MXE, *CDC6* is the only gene significantly upregulated in the cell cycle pathway. Therefore, we chose *CDC6* for further analysis.

*CDC6* is located on 17q21.2 (40,287,878–40,304,657). It is involved in the initiation of DNA replication and checkpoint controls that ensure DNA replication is completed before mitosis is initiated. The FPKM levels of the six transcripts for CDC6 were listed in Additional File 11: Table S9. We chose ENST00000209728 for further study, which was detectable in all of the samples and was significantly upregulated in SLE patients. The qRT-PCR verification results of ENST00000209728 were shown in Fig. 5c. We identified MXE (chr17: 40,295,355–40,302,236) of *CDC6* in the PBMCs (Fig. 6b). Exon 2 is abundant in the SLE group and is absent in the control group. Figure 6c showed the Sashimi Plot of the MXE events in the *CDC6* of CTRL1 and SLE19 samples. In the Junction Track of IGV, radians between histograms display splice junctions detected in reads. The junctions reads of SLE19 were significantly higher than that of CTRL1 on exon 2. Therefore, *CDC6* has more MXE may explain the significant upregulation of *CDC6* in SLE patients.

**Figure 7:**
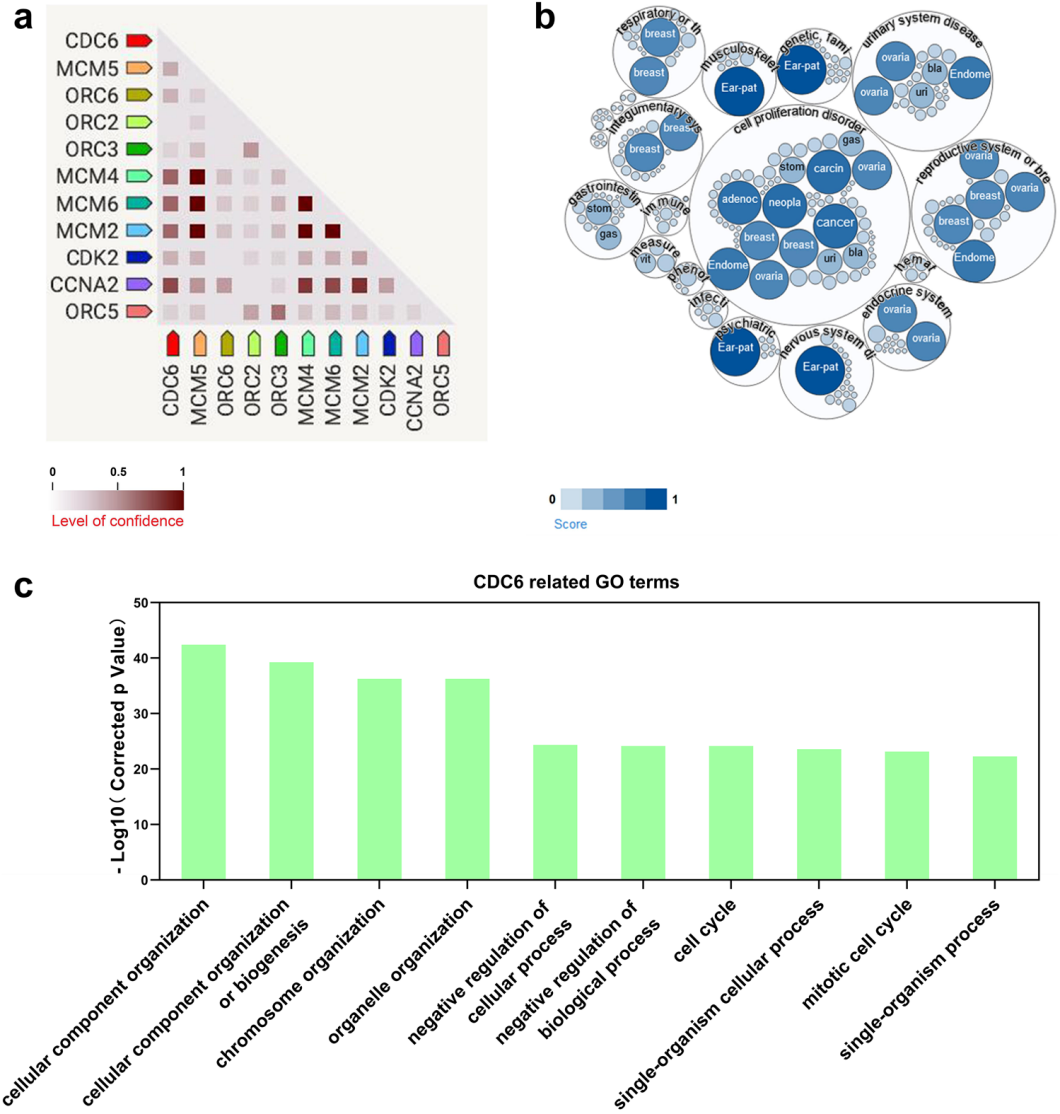
Protein–protein interactions and the roles of cell division cycle 6 (*CDC6*) in diseases. **(a)** Diagram of the top 10 factors interacting with *CDC6*. **(b)** Enrichment diagram of *CDC6*-related diseases. The depth of the color represents the degree of correlation. **(c)** The top ten dysregulated GO terms associated with *CDC6* in the systemic lupus erythematosus (SLE) group compared to the control (CTRL) group.

We further analyzed the protein–protein interaction network associated with *CDC6* (Fig. 7a). Results showed that *CDC6* was closely related to *MCM2*, *MCM4*, *MCM6*, or *CCNA2*. According to published studies, *CDC6* is involved in 107 diseases as shown in the disease enrichment map (Fig. 7b). Cell proliferation disorders (Association score: 0.82, 64 diseases) are the most common causes of *CDC6*-related diseases. *CDC6* is also involved in urinary system disease (Association score: 0.79, 23 diseases) and respiratory or thoracic disease (Association score: 0.79, 21 diseases). Ear-patella-short stature syndrome (Association score: 1.00) is a very rare genetic disorder that is also strongly associated with *CDC6*. We further found that *CDC6*-related GO terms were significantly upregulated in the SLE group (Fig. 7c), and these GO terms are related to cell cycle and cellular components.

**Figure 8:**
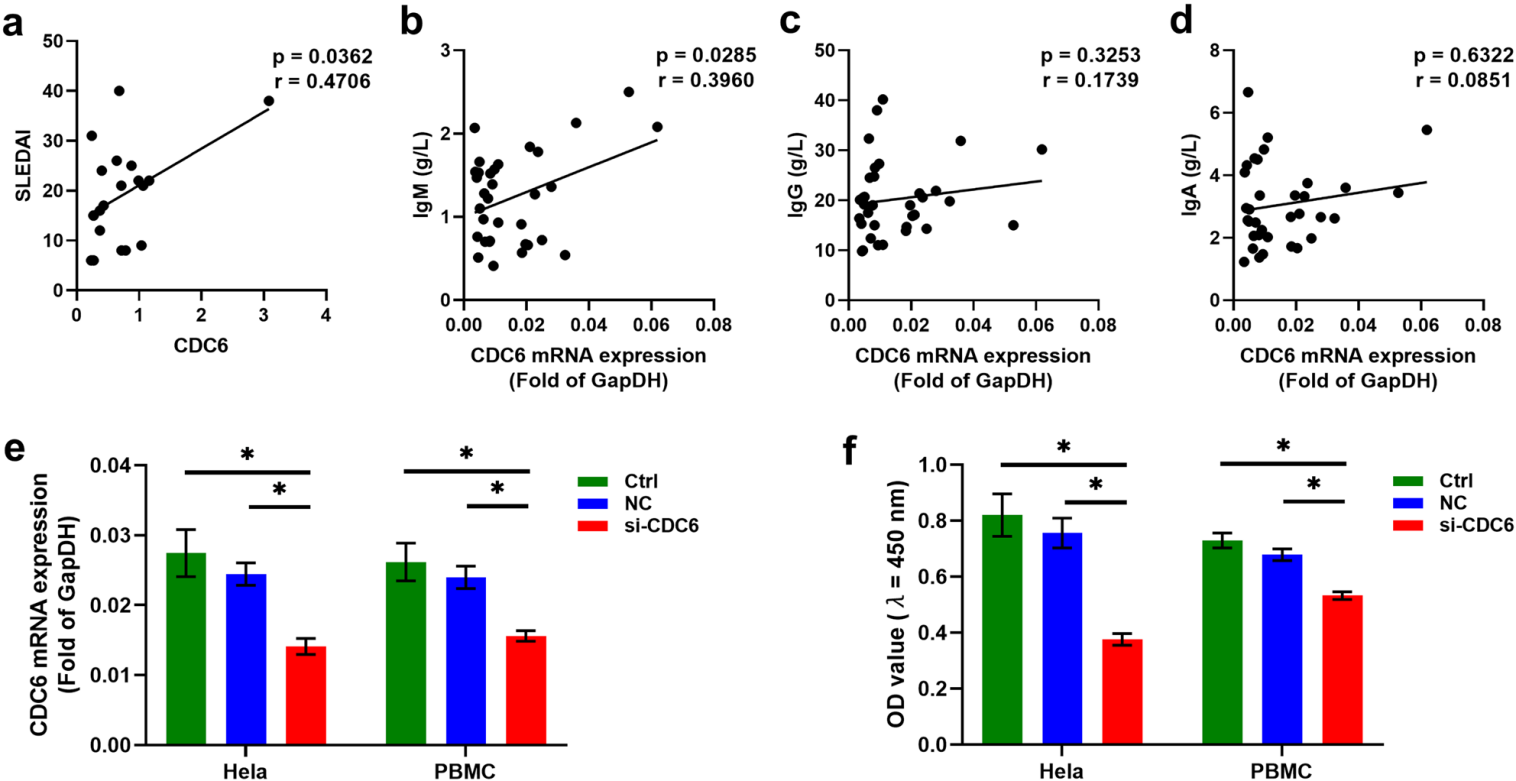
Analysis of the relationship between cell division cycle 6 (*CDC6*) and SLE disease. **(a)** Correlations between the *CDC6* levels and the SLEDAI of SLE patients. **(b)** Correlations between the *CDC6* levels and the IgM levels in SLE patients. **(c)** Correlations between the *CDC6* levels and the IgG levels in SLE patients. **(d)** Correlations between the *CDC6* levels and the IgA levels in SLE patients. **(e)** *CDC6* expression in Hela cells and SLE patients’ PBMCs was detected by Quantitative real-time PCR (qRT-PCR). **(f)** The effect of si-*CDC6* on cell proliferation was analyzed by cell counting kit-8 (CCK-8) assay. *P < 0.05.

We analyzed the relationship between *CDC6* level and patient clinical data. Results showed that FPKMs of *CDC6* (ENST00006209728) were positively correlated with SLEDAI levels in SLE patients (Fig. 8a), which indicates that *CDC6* may play an important role in the pathogenesis of SLE. In addition, the analysis of expanded samples of PBMCs from SLE patients showed that the expression of *CDC6* was positively correlated with the level of IgM antibody in the blood of SLE patients (Fig. 8b). However, no obvious correlations were found between *CDC6* levels and IgG or IgA levels in SLE patients (Fig. 8c, d). Therefore, we speculate that *CDC6* may affect the production of IgM antibodies.

As shown in Additional File 12: Figure S3, the results from the single-cell sequencing database suggested that *CDC6* was mainly expressed in memory B cells, naive B cells, T cells, and NK cells. We further analyzed our sequencing data on cell proportions in PBMCs of SLE patients or healthy controls (Additional File 13: Figure S4). The proportion of naive B cells, plasma cells, and monocytes was significantly upregulated, whereas the proportion of T cells, NK cells, and DC cells was significantly downregulated, in patients with SLE compared with controls. These results indicate that naive B cells and plasma cells are highly proliferative cells in PBMCs of SLE patients. In order to explore the function of *CDC6* in human cells and in SLE, we transfected Hela cells or SLE patients' PBMCs with si-*CDC6* mRNA, which significantly inhibited the expression of *CDC6* in these cells (Fig. 8e) After *CDC6* knockdown, the proliferation of Hela cells and PBMCs were prominently impaired compared with si-NC or Control (Fig. 8f). These data suggested that *CDC6* knockdown inhibited human cell proliferation in SLE in vitro.

## Discussion

The pathogenesis of SLE is incompletely understood. We investigated the key pathways and factors involved in the pathogenesis of SLE by high-throughput sequencing to provide a molecular basis for effective treatment of SLE.

We identified 1494 upregulated and 1001 downregulated genes in SLE patients compared to controls. The most significantly upregulated pathway is the SLE pathway, which is easy to understand and similar to some reports^32^. We found that in patients with SLE: (1) Histone (*H2B*, *H3*, *H4*) and complements (*C1q*, *C2*) are significantly upregulated to participate in antigen presentation; (2) *HLA-DPA1* and *IL-10* are significantly upregulated to participate in cell activation; (3) *C1q*, *C2*, *CSTG*, *ELANE*, and *FcγR* are significantly upregulated and involved in tissue injury. Mo et al*.* also reported significantly upregulated SLE pathway in SLE patients^33^. We found that *FcγR* is significantly upregulated in SLE patients. Similarly, Zhang et al*.* suggested that the most differentially expressed genes were enriched on the *FcγR*-mediated phagocytosis pathway^34^. On the other hand, Ye et al*.* studied the targeted mRNA of long-chain non-coding RNA in SLE patients, but did not mention the SLE pathway in their enrichment analysis^16^.

Genes significantly upregulated in SLE patients were enriched on the GO terms related to cell cycle, stress response, and chromosome organization. The 235 upregulated genes enriched in the cell cycle term, belonged to the *CDC* family, *E2F* family, *MCM* family, and *ORC* family. Similarly, Ye et al*.* screened 740 mRNAs targeted by 23 lincRNAs in SLE patients. These genes were mainly involved in DNA binding and transcription, cell cycle, etc.^16^, which was mutually confirmed by our results. In addition, we found that 37 differently expressed genes affecting DNA replication were enriched in the G1-S phase of the cell cycle pathway. We validated some upregulated genes (*E2F1*, *E2F2*, *ORC1*, *ORC6*, *MCM2*, *MCM4*, *CDC6*, *CDC45*) and found close protein interactions between these factors. In particular, the interaction between *CDC6* and *MCM2,4,6* was highly reliable. Differently, Zhang et al*.* demonstrated disregulated GO terms of ribonucleotide, protein serine/threonine kinase activity function, and regulation of B cell differentiation in SLE patients^34^. Zhang et al*.* used whole blood (3 samples in each group) whereas we used PBMCs (10 or 20 samples in each group). These sampling differences may partly explain our different results.

Regulation of the cell cycle is a basic biological process that ensures the function and health of the entire organism^35^. Hu et al*.* reported the prognostic and diagnostic value of the increased expression of *CDC6*, *CDC45*, and *ORC6* in the cell cycle pathway in colorectal cancer^36^. The occurrence of ear-patella-short syndrome is related to genetic mutations in *CDC6*, *ORC1*, *ORC4*, *ORC6*, and *CDT1*^37–39^. SLE patients have a high proportion of low-density neutrophils (LDNs)^40^. The upregulation of the cell cycle pathway in CD10^-^ LDNs leads to the immaturity of these cells^41^. Gao et al*.* found that the cell cycle pathway may be involved in the pathogenesis of nephritis in SLE patients^42^. Nafise Tabasi et al*.* believe that Vitamin D inhibits cell cycle progression, which regulates and stabiliz the immune system of SLE patients^43^.

*CDC6* is involved in the formation of pre-replication complexes (pre-RC), which play a key role in the regulation of DNA replication and cell proliferation^44–46^. *CDC6* is not only required for G1 origin licensing, but is also crucial for proper S-phase DNA replication that is essential for DNA segregation during mitosis^47^ *CDC6* is a specific biomarker of proliferating cells^48–50^, and a candidate prognostic marker associated with cell apoptosis and cell cycle^51^. *CDC6* overexpression interferes with the expression of INK4/ARF tumor suppressor genes through a mechanism involving the epigenetic modification of chromatin at the INK4/ARF locus^52^. Therefore, *CDC6* overexpression is a feature of tumors and is associated with early events of malignancies^53–56^. We found highly expressed *CDC6* in the peripheral blood of SLE, which may increase DNA replication and cell proliferation as a key factor in the pathogenesis of SLE. People found that the transcription factor binding sites of *E2F1* were significantly over-represented in SLE loci^57^, while drugs targeting *E2F1* lead to innovative therapies for SLE^58^. We suppose *CDC6* may become an effective and innovative target for the treatment of SLE as well.

Research on alternative splicing attracted more and more attention^59–61^. People reported increased alternative splicing events for *IRF5* which was associated with the incidence and severity of lupus^62^. Moulton et al*.* reported that the selective splicing of *CD3ζ* partly downregulated the levels of *CD3ζ* in patients with SLE, which in turn downregulated T cell function^63–65^. We revealed that the MXE levels of *CDC6* in the SLE group were significantly higher than that of the control group, which may contribute to the high expression of *CDC6* in SLE patients.

Furthermore, we found that the level of *CDC6* was significantly correlated with the level of SLEDAI or IgM, and the knockdown of *CDC6* in PBMCs from SLE patients inhibited the proliferation of these cells. Therefore, *CDC6* plays an important role in the proliferation of human cells and the pathogenesis of SLE. The cell cycle pathway has been used as the therapeutic target of cancer^66–72^. *CDC6* expression in glioma was positively correlated with Th2 cells, Macrophages and Eosinophils, and negatively correlated with plasmacytoid dendritic cells, CD8 T cells and NK CD56bright cells, suggesting its role in regulating tumor immunity. MAPK pathway, P53 pathway, and NF-κB pathway in cancer were differentially enriched with high *CDC6* expression. Silencing *CDC6* could significantly inhibit proliferation, migration, invasion, and promoted apoptosis of U87 cells and U251 cells^73^. Cyclophosphamide inhibits proliferation of mesangial cells by downregulating cell cycle regulator including cyclins and cyclin-dependent kinases and therefore arresting them at G1 phase^74^. People identified *Cdkn2c*, a gene that controls cell cycle progression, as a key regulator of B-1a cell numbers. *Cdkn2c* deficiency associated with SLE pathogenesis, including the production of autoantibodies and the skewing of CD4( +) T cells toward inflammatory effector functions^75^. APRIL is reported to modify genes specifically related to cell cycle modulation, including *CDC6*^76^. We speculate that *CDC6* may be an effective target for SLE. Inhibition of *CDC6* in hyperproliferative B may become an effective treatment regimen for SLE.

The current study has several limitations. Firstly, we performed a high-throughput sequencing analysis on PBMCs but did not further determine the cell type. The situation for *CDC6* in B cells from SLE patients is unclear. However, it is found that the G1321A polymorphism of *CDC6* was associated with decreased risk for B-cell-non-Hodgkin lymphoma, which suggested the role of *CDC6* in B cells^77^. Normally, *CDC6* was mostly expressed in memory B cells, naive B cells, T cells, and NK cells of PBMCs. Our data suggested the proportions of naive B cells, plasma cells, and monocytes in PBMCs were significantly upregulated in SLE patients compared to controls. SLE is considered an autoimmune disease of B cell hyperactivity^7^. Thus, we hypothesized that the enhancement of *CDC6* expression and the activation of the cell cycle pathway in SLE patients are likely to occur at least in B cells. Secondly, the relationship between *CDC6* and LN is not clear. We didn’t find correlation between *CDC6* expression levels in PBMCs and urinary protein levels. Hence, no evidence can be provided so far to support the association of *CDC6* with lupus nephritis.

## Conclusions

We identified 2495 differently expressed genes for SLE. The SLE pathway, cell cycle pathway, and key factor *CDC6* were significantly upregulated. We revealed upregulated MXE alternative splicing events in the *CDC6* of SLE patients, which may lead to its upregulation in these patients. The expression of *CDC6* was positively correlated with SLEDAI level or IgM level in SLE patients. Knockdown of *CDC6* suppressed the proliferation of SLE patients’ PBMCs in vitro, hinting that *CDC6* participated in the pathological proliferation of SLE. These findings may provide new ideas for the effective treatment of SLE.

## Supplementary Information

Supplementary Information.

## Data Availability

The datasets generated and/or analyzed during the current study are available in the GEO repository (accession number: GSE211700, https://www.ncbi.nlm.nih.gov/geo/query/acc.cgi?acc=GSE211700, token number: efalyswybxgthmz).

## Abbreviations

ACR: american college of rheumatology
BP: biological process
CC: cellular component
CCK-8: cell counting kit-8
CTRL: control
GO: gene ontology
GWAS: genome-wide association studies
KEGG: kyoto encyclopedia of genes and genomes
LDNs: low-density neutrophils
MF: molecular function
MXE: mutual exclusive exon
PBMCs: peripheral blood mononuclear cells
pre-RC: pre-replication complexes
PCA: principal component analysis
PPI: protein–protein interaction
qRT-PCR: quantitative real-time PCR
RA: rheumatoid arthritis
RYBP: RING1 and YY1-Binding protein
si-RNA: small interference RNA
SLE: systematic lupus erythematosus
