# Supplementary material for: High throughput sequencing revealed enhanced cell cycle signaling in SLE patients
Source: Sci Rep. 2023 Jan 4;13:159. doi: 10.1038/s41598-022-27310-8 (PMC9812989; doi:10.1038/s41598-022-27310-8)
Supplement: Supplementary file 1 — Supplementary Information. [file 41598_2022_27310_MOESM1_ESM.pdf]

High throughput sequencing revealed enhanced cell cycle signaling in SLE patients

Mingyue Yang[a¶], Peisong Wang[b¶], Tao Liu[c], Xiaojuan Zou[c], Ying Xia[a], Chenxu Li[a], Xiaosong Wang[a*]

a. Laboratory for Tumor Immunology, Translational Medicine Department, First Hospital of Jilin University, Changchun, 130021, China

b. Thyroid Surgery Department, General Surgery Center, First Hospital of Jilin University Changchun, 130021, China

c. Department of Rheumatology and Immunology, First Hospital of Jilin University, Changchun, 130021, China

*** Correspondence author:**

Xiaosong Wang

Present address: Institute of Translational Medicine, First Hospital of Jilin University, No.519 Dongminzhu Street, Changchun 130021, China

+86 0431-88783044

E-Mail: xiaosongwang@jlu.edu.cn

¶ These authors have contributed equally to this work and share the first authorship

Additional File 1: Table S1. Clinical information for the sequencing samples

	Ctrl (*n* = 10)	SLE (*n* = 20)	*P* value
**Gender (female/male)**	10/0	19/1	0.5245
**Median age (range), years**	31 (24 - 37)	36 (15 - 67)	0.2257
**SLEDAI**	-	19.10 ± 9.88	-
**Proteinuria, g/24 hour**	-	0.62 (0.235 - 2.845)	-
**IgG [7-16 (g/L)]**	-	23.07 ± 12.00	-
**IgA [0.7-4 (g/L)]**	-	3.88 ± 1.41	-
**IgM [0.4-2.3 (g/L)]**	-	1.11 (0.96 - 1.49)	-
**Anti-dsDNA antibody-positive, %**	-	90	-
**Complement C3, g/L**	-	0.57 ± 0.29	-

Quantitative data with a normal distribution are presented as mean ± *SD*. Quantitative data with a non-normal distribution are presented as median (IQR).

Additional File 2: Table S2. Clinical information for the qRT-PCR and function asssay samples

	Ctrl (n = 40)	SLE (n = 58)	P value
**Gender (female/male)**	37/3	53/5	0.846
**Median age (range), years**	49 (20 - 72)	37.5 (19 - 67)	0.023*
**SLEDAI**	-	14.49 ± 7.46	-
**Proteinuria, g/24 hour**	-	0.31 (0.19 - 1.40)	-
**IgG [7-16 (g/L)]**	-	18.4 (15 - 21.9)	-
**IgA [0.7-4 (g/L)]**	-	2.91 (2.09 - 3.75)	-
**IgM [0.4-2.3 (g/L)]**	-	1.26 (0.75 - 1.57)	-
**Anti-dsDNA antibody-positive, %**	-	75.8	-
**Complement C3, g/L**	-	0.50 (0.29 - 0.63)	-

Quantitative data with a normal distribution are presented as mean ± *SD*. Quantitative data with a non-normal distribution are presented as median (IQR). * represent p <0.05.

Additional File 3: Table S3. The top 20 upregulated genes of SLE patients compared to controls

**Transcript_id**	**Gene_id**	**Official_Symbol**	**Readcount_SLE**	**Readcount_Control**	**log2FoldChange**	***P* value**	***Q* value**
ENST00000369798	ENSG00000112742	*TTK*	5.448883	0.031658	7.176844	2.83E-19	1.03E-14
ENST00000538992	ENSG00000010030	*ETV7*	1.932543	0	8.995962	1.47E-17	3.19E-13
ENST00000251496	ENSG00000109805	*NCAPG*	21.80773	1.706339	3.664139	1.61E-16	2.92E-12
ENST00000423485	ENSG00000131747	*TOP2A*	56.46557	7.473511	2.952885	1.18E-15	1.83E-11
ENST00000367409	ENSG00000066279	*ASPM*	37.03352	4.430091	3.087378	7.25E-15	9.85E-11
ENST00000370747	ENSG00000137965	*IFI44*	348.9391	54.01427	2.726296	8.39E-15	1.01E-10
ENST00000247191	ENSG00000126787	*DLGAP5*	10.23233	0.806999	3.693811	4.4E-14	3.64E-10
ENST00000359303	ENSG00000197153	*HIST1H3J*	39.55995	3.614961	3.45468	4.5E-14	3.64E-10
ENST00000614378	ENSG00000273983	*HIST1H3G*	73.74846	6.265994	3.61338	4.69E-14	3.64E-10
ENST00000295934	ENSG00000163666	*HESX1*	1.803424	0	8.956164	8.58E-14	5.83E-10
ENST00000301905	ENSG00000168078	*PBK*	2.878755	0.174212	4.05527	1.12E-13	7.14E-10
ENST00000215794	ENSG00000184979	*USP18*	58.51551	6.082503	3.284799	1.41E-13	7.65E-10
ENST00000370751	ENSG00000137959	*IFI44L*	855.4926	87.11009	3.324355	1.28E-13	7.65E-10
ENST00000612966	ENSG00000278272	*HIST1H3C*	61.1199	6.392374	3.283293	1.37E-13	7.65E-10
ENST00000377459	ENSG00000274997	*HIST1H2AH*	56.55196	6.933696	3.046091	2.64E-13	1.25E-09
ENST00000578186	ENSG00000270882	*HIST2H4A*	120.8117	18.14651	2.733286	6.4E-13	2.57E-09
ENST00000621411	ENSG00000274267	*HIST1H3B*	117.1782	13.84238	3.114458	6.67E-13	2.59E-09
ENST00000618200	ENSG00000165949	*IFI27*	18.48989	0.052872	8.272746	9.79E-13	3.67E-09
ENST00000619466	ENSG00000275713	*HIST1H2BH*	60.65081	9.450497	2.711019	1.21E-12	4.23E-09
ENST00000331442	ENSG00000184357	*HIST1H1B*	189.124	25.64664	2.933392	1.25E-12	4.23E-09

Additional File 4: Table S4. The top 20 downregulated genes of SLE patients compared to controls

Transcript_id	Gene_id	Official_Symbol	Readcount_SLE	Readcount_Control	log2FoldChange	*P* value	*Q* value
ENST00000431380	ENSG00000159674	*SPON2*	0.120543	27.15965	-7.72812	5.70E-24	6.20E-19
ENST00000377988	ENSG00000137103	*TMEM8B*	0	2.599678	-9.50353	2.21E-23	1.20E-18
ENST00000615871	ENSG00000158321	*AUTS2*	0.004472	6.260874	-9.84135	6.37E-19	1.73E-14
ENST00000368678	ENSG00000010810	*FYN*	0	62.20345	-13.9857	2.92E-14	2.89E-10
ENST00000330232	ENSG00000093072	*ADA2*	0.278645	32.51303	-6.35484	6.98E-14	5.05E-10
ENST00000406733	ENSG00000054611	*TBC1D22A*	0.32859	5.841493	-4.0291	1.82E-13	9.44E-10
ENST00000611706	ENSG00000158321	*AUTS2*	0.138878	7.666615	-5.69785	4.72E-13	2.05E-09
ENST00000590214	ENSG00000180448	*ARHGAP45*	3.15924	30.76977	-3.24665	4.13E-12	1.07E-08
ENST00000524950	ENSG00000120458	*MSANTD2*	0	0.62121	-7.51957	9.65E-12	2.19E-08
ENST00000426154	ENSG00000136193	*SCRN1*	0	7.508914	-11.1222	1.22E-10	1.96E-07
ENST00000620073	ENSG00000096384	*HSP90AB1*	0.051842	1.296375	-4.57348	1.36E-10	2.11E-07
ENST00000428334	ENSG00000157985	*AGAP1*	0.044114	3.19111	-6.0308	2.93E-10	4.03E-07
ENST00000309190	ENSG00000153113	*CAST*	4.339847	46.98413	-3.45703	3.20E-10	4.35E-07
ENST00000300527	ENSG00000142173	*COL6A2*	8.445153	36.33145	-2.02146	3.66E-10	4.76E-07
ENST00000605057	ENSG00000100813	*ACIN1*	0.46775	8.595377	-4.51071	4.87E-10	5.94E-07
ENST00000642274	ENSG00000011376	*LARS2*	0.356736	3.950788	-3.35256	1.47E-09	1.44E-06
ENST00000361390	ENSG00000198888	*MT-ND1*	76.33276	313.1434	-2.01242	1.56E-09	1.48E-06
ENST00000430193	ENSG00000104835	*SARS2*	0.05786	1.107996	-4.09424	2.80E-09	2.38E-06
ENST00000464606	ENSG00000105939	*ZC3HAV1*	34.83475	127.4275	-1.76093	3.22E-09	2.63E-06
ENST00000373988	ENSG00000147130	*ZMYM3*	0.007282	4.10235	-8.18719	3.37E-09	2.73E-06

Additional File 5: Table S5. Top 30 significantly upregulated GO terms for SLE patients

GO_accession	Description	Term_type	Over_represented_*P* Value	Corrected_*P* Value
GO:0044446	intracellular organelle part	cellular_component	1.55E-51	3.32E-47
GO:0044422	organelle part	cellular_component	1.37E-50	1.47E-46
GO:0005694	chromosome	cellular_component	1.10E-49	7.84E-46
GO:0005515	protein binding	molecular_function	6.93E-47	3.71E-43
GO:0044424	intracellular part	cellular_component	2.13E-46	8.30E-43
GO:0044815	DNA packaging complex	cellular_component	2.70E-46	8.30E-43
GO:0043233	organelle lumen	cellular_component	2.71E-46	8.30E-43
GO:0044427	chromosomal part	cellular_component	1.06E-45	2.85E-42
GO:0031974	membrane-enclosed lumen	cellular_component	1.22E-45	2.90E-42
GO:0005622	intracellular	cellular_component	9.09E-45	1.95E-41
GO:0051276	chromosome organization	biological_process	8.04E-44	1.57E-40
GO:0043231	intracellular membrane-bounded organelle	cellular_component	3.17E-43	5.65E-40
GO:0043227	membrane-bounded organelle	cellular_component	2.32E-41	3.83E-38
GO:0000786	nucleosome	cellular_component	6.32E-41	9.66E-38
GO:0006996	organelle organization	biological_process	2.16E-40	3.08E-37
GO:0043229	intracellular organelle	cellular_component	4.04E-40	5.41E-37
GO:0070013	intracellular organelle lumen	cellular_component	7.03E-40	8.85E-37
GO:0043226	organelle	cellular_component	3.48E-39	4.14E-36
GO:0071103	DNA conformation change	biological_process	8.61E-37	9.70E-34
GO:0007049	cell cycle	biological_process	1.52E-36	1.63E-33
GO:0005737	cytoplasm	cellular_component	2.25E-36	2.29E-33
GO:0044444	cytoplasmic part	cellular_component	3.88E-35	3.77E-32
GO:0006950	response to stress	biological_process	4.96E-35	4.61E-32
GO:0000278	mitotic cell cycle	biological_process	2.21E-33	1.97E-30
GO:0032993	protein-DNA complex	cellular_component	5.05E-33	4.32E-30
GO:0044428	nuclear part	cellular_component	7.75E-33	6.38E-30

GO:0051707	response to other organism	biological_process	9.03E-33	7.16E-30
GO:0016043	cellular component organization	biological_process	9.86E-33	7.54E-30
GO:0043207	response to external biotic stimulus	biological_process	1.10E-32	8.10E-30
GO:0031981	nuclear lumen	cellular_component	2.84E-32	2.03E-29

Additional File 6: Table S6. Top 30 significantly downregulated GO terms for SLE patients

GO_accession	Description	Term_type	Over_represented_*P* Value	Corrected_*P* Value
GO:0005515	protein binding	molecular_function	6.23E-16	1.33E-11
GO:0016043	cellular component organization	biological_process	4.47E-15	4.26E-11
GO:0032502	developmental process	biological_process	6.01E-15	4.26E-11
GO:0048856	anatomical structure development	biological_process	7.97E-15	4.26E-11
GO:0044767	single-organism developmental process	biological_process	3.52E-14	1.51E-10
GO:0044424	intracellular part	cellular_component	5.46E-14	1.95E-10
GO:0071840	cellular component organization or biogenesis	biological_process	9.43E-14	2.89E-10
GO:0005622	intracellular	cellular_component	1.17E-13	3.13E-10
GO:0007275	multicellular organismal development	biological_process	1.68E-13	4.00E-10
GO:0005488	binding	molecular_function	2.61E-13	5.58E-10
GO:0048731	system development	biological_process	3.14E-13	6.11E-10
GO:0009653	anatomical structure morphogenesis	biological_process	3.59E-13	6.41E-10
GO:0000902	cell morphogenesis	biological_process	1.22E-12	2.00E-09
GO:0032989	cellular component morphogenesis	biological_process	1.40E-12	2.14E-09
GO:0023051	regulation of signaling	biological_process	1.84E-12	2.58E-09
GO:0009966	regulation of signal transduction	biological_process	1.93E-12	2.58E-09
GO:0010646	regulation of cell communication	biological_process	2.20E-12	2.76E-09
GO:0000122	negative regulation of transcription from RNA polymerase II promoter	biological_process	2.42E-12	2.76E-09
GO:0007399	nervous system development	biological_process	2.45E-12	2.76E-09
GO:0000904	cell morphogenesis involved in differentiation	biological_process	2.89E-12	3.09E-09
GO:0048523	negative regulation of cellular process	biological_process	4.78E-12	4.87E-09
GO:0048468	cell development	biological_process	5.14E-12	5.00E-09
GO:0048869	cellular developmental process	biological_process	1.08E-11	1.01E-08
GO:0048583	regulation of response to stimulus	biological_process	1.36E-11	1.21E-08
GO:0048518	positive regulation of biological process	biological_process	1.43E-11	1.22E-08

GO:0050793	regulation of developmental process	biological_process	1.52E-11	1.25E-08
GO:0048519	negative regulation of biological process	biological_process	1.59E-11	1.26E-08
GO:0051128	regulation of cellular component organization	biological_process	1.83E-11	1.40E-08
GO:0006357	regulation of transcription from RNA polymerase II promoter	biological_process	2.53E-11	1.87E-08
GO:0030154	cell differentiation	biological_process	6.72E-11	4.77E-08

Additional File 7: Table S7. Top 20 downregulated KEGG pathways for SLE patients

pathway_term	rich_factor	qvalue	gene_number
Circadian rhythm	0.258064516	0.150819282	8
Wnt signaling pathway	0.114285714	0.317294248	16
Hippo signaling pathway	0.11038961	0.317294248	17
Natural killer cell mediated cytotoxicity	0.111940299	0.317294248	15
Adherens junction	0.136986301	0.317294248	10
Colorectal cancer	0.14516129	0.317294248	9
Lysine degradation	0.156862745	0.317294248	8
Vasopressin-regulated water reabsorption	0.155555556	0.409323135	7
Osteoclast differentiation	0.106870229	0.409323135	14
Focal adhesion	0.09178744	0.451160905	19
Malaria	0.142857143	0.451160905	7
Arrhythmogenic right ventricular cardiomyopathy (ARVC)	0.121621622	0.451160905	9
Melanogenesis	0.108910891	0.451160905	11
Thyroid cancer	0.172413793	0.451160905	5
T cell receptor signaling pathway	0.105769231	0.473454959	11
Antigen processing and presentation	0.113924051	0.473454959	9
Thyroid hormone signaling pathway	0.100840336	0.473454959	12
Basal cell carcinoma	0.127272727	0.476063764	7
Hypertrophic cardiomyopathy (HCM)	0.108433735	0.534843431	9
Endocytosis	0.084507042	0.555523814	18

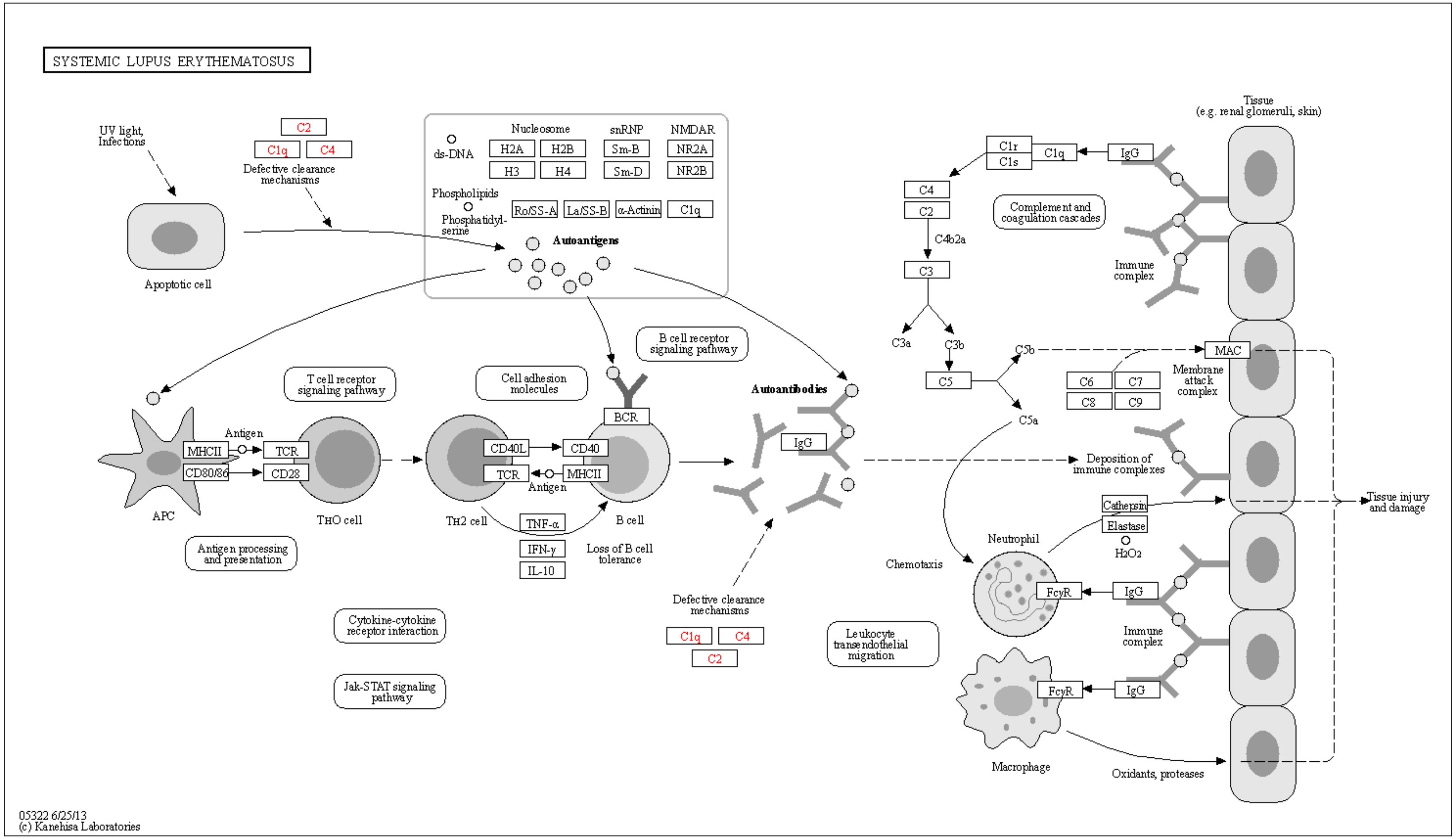

Additional File 8: Figure S1. The map of systemic lupus erythematosus pathway from KEGG's website (KEGG PATHWAY: map05322)

Additional File 9: Table S8. Detailed information of the differential genes in the pathways

Pathway	Gene name
SLE	HIST1H3J***, HIST1H4A*, HIST1H4C**, C2*, HIST1H4H***, HIST2H4B***, HIST1H4L***, HIST1H2BM***, HIST1H2BJ***, HIST1H2BN***, HIST1H2BH***, ELANE***, CTSG***, HIST2H2BF*, HIST1H2BO***, IL10***, FCGR1A***, HIST1H2BE*, C1QB**, HIST2H2AA4***, HLA-DPA1*, C1QC**, HIST1H4K***, HIST3H2BB***, HIST2H2AA3***, HIST2H3C***, HIST1H3G***, HIST1H2BF***, HIST1H4J**, HIST1H2AH***, HIST2H3A***, HIST1H4B*, HIST1H2BG***, HIST1H2BD***, HIST1H2AI***, HLA-DOB*, HIST1H2AG***, HIST1H3B***, HIST2H3D***, HIST1H2AJ***, HIST1H3C***, HIST1H2BK***, HIST1H4F*, HIST1H2BI***, HIST1H2BL***, HIST1H2AL***, HIST1H2AE**, HIST1H4D***, H2AFZ*, HIST1H2BB***, HIST1H2AB***, HIST3H2A**, HIST2H2AB*, HIST2H4A***, H2AFJ**, HIST1H3H***, HIST2H2BE***, C1QA**, HIST4H4*, CD40LG*, HIST1H3F***, HIST1H4I***, HIST1H2AC*, FCGR1B***, HIST1H2AM***
Cell cycle	RBX1**, E2F3***, CHEK1***, BUB1***, CDC25C***, PTTG1**, TGFB1**, CCNA2***, ESPL1*, RB1*, PKMYT1**, PLK1***, CDC25A***, CCNB2***, CCNE2**, MAD2L1**, E2F2***, CDC20*, E2F1***, MAD2L2**, MDM2*, PCNA***, CREBBP*, MCM2**, ORC4*, MCM4***, BUB1B***, ORC6*, ORC1**, TTK***, TFDP2*, CDC45***, CCNB1**, SMAD4**, CDC6***, YWHAH**, CDKN2C*

* corrected P <0.05, ** corrected P <0.01, *** corrected P <0.001.

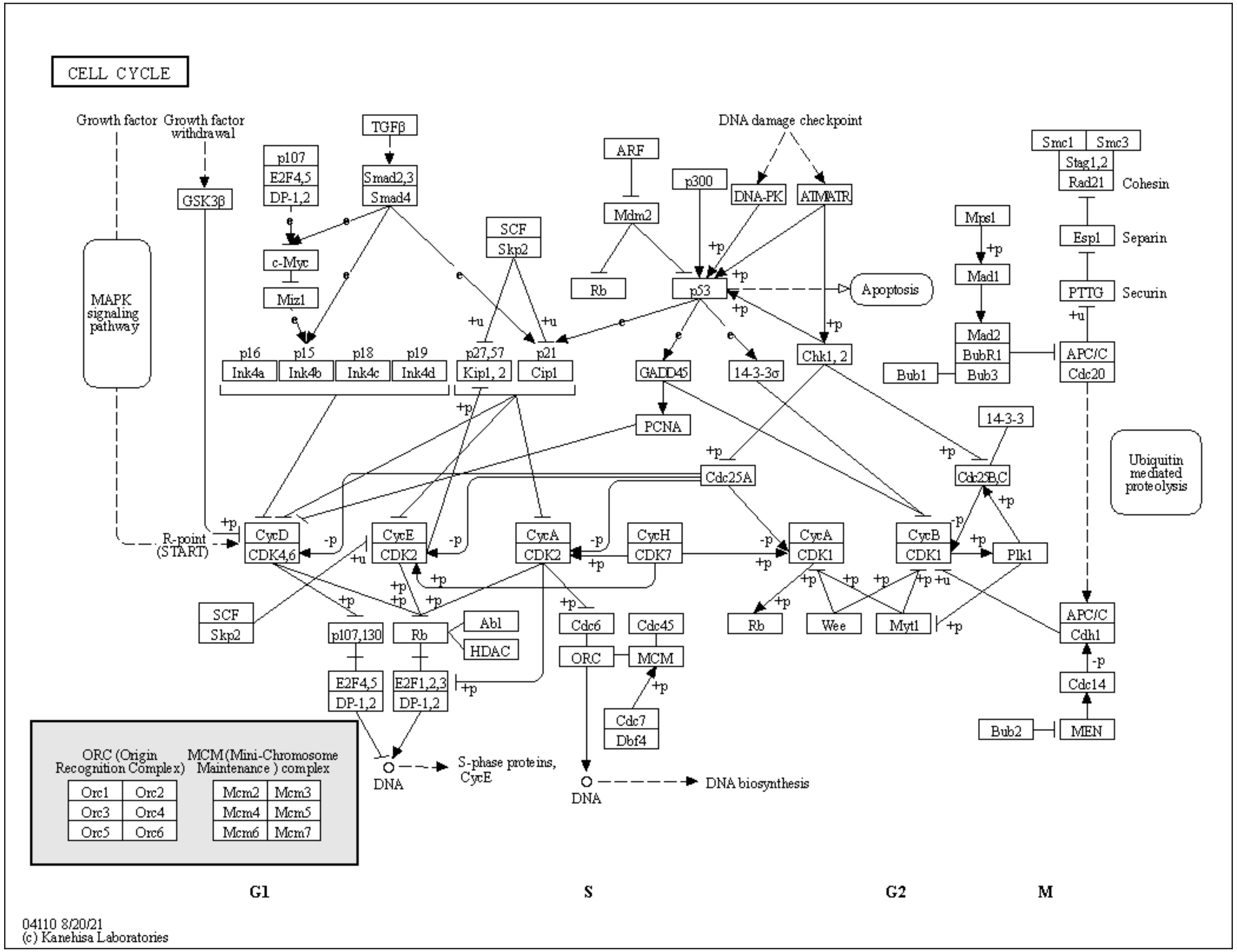

Additional File 10: Figure S2. The map of cell cycle pathway from KEGG's website (KEGG PATHWAY: map04110 (genome.jp)).

Additional File 11: Table S9. The sequencing results of different transcripts for *CDC6*

Transcript_id	qvalue	CTRL_FPKMs										SLE_FPKMs																			
ENST00000647931	6.23E-08	0	0	0	0	0	0	0	0.068146	0	0	0.824585	1.366428	0.694319	2.100392	1.646166	1.896326	4.666018	1.811975	5.438606	1.838658	0.92683	1.316293	0.88411	1.450761	3.144522	0.619898	0	1.9932	0	0
ENST00000209728	0.00206	0.159785	0.172488	0.416652	0.183727	0.091461	0.17747	0.155377	0.242134	0.263326	0.250967	0.242696	3.08213	0.640464	1.065426	0.401211	0.370207	0.680384	0.269543	0.715443	0.879403	0.71963	0.992953	1.161671	0.271213	0.433028	0.273327	0.784232	0.370348	0.231805	1.03667
ENST00000577249	0.062394	0	0	0	0	0	0	0	0	0	0.121249	0.202723	0.346117	0	0.15004	0.014411	0.112975	0.130916	0	0.080913	0.133334	0.066151	0.101308	0.008269	0	0	0.070782	0	0.090063	0.187995	0.060624
ENST00000649662	0.17281	0	0	0	0	0	0	0	0	0	0	0	0	0	0	0	0	0	0	0	7.969693	0	0	0	0	0	0	0	0	0	0
ENST00000580824	0.39368	0	0	0	0	0	0	0	0	0	0	0	0	0	0.392795	0	0	0	0	0	0	0.217687	0.330488	0.344146	0	0	0	0	0	0	0
ENST00000473555	0.544745	0.163005	0.187001	0.503964	0.320914	0.234767	0.221902	0.314275	0.002993	0.32707	0.133576	0.12084	0.572561	0.219691	0.696963	1.114948	0.244851	1.540589	0.003745	0.054453	0.470545	0.325307	0.494949	0.817278	0.01997	0.155242	0.040636	0.953065	0.573028	0	0.401288

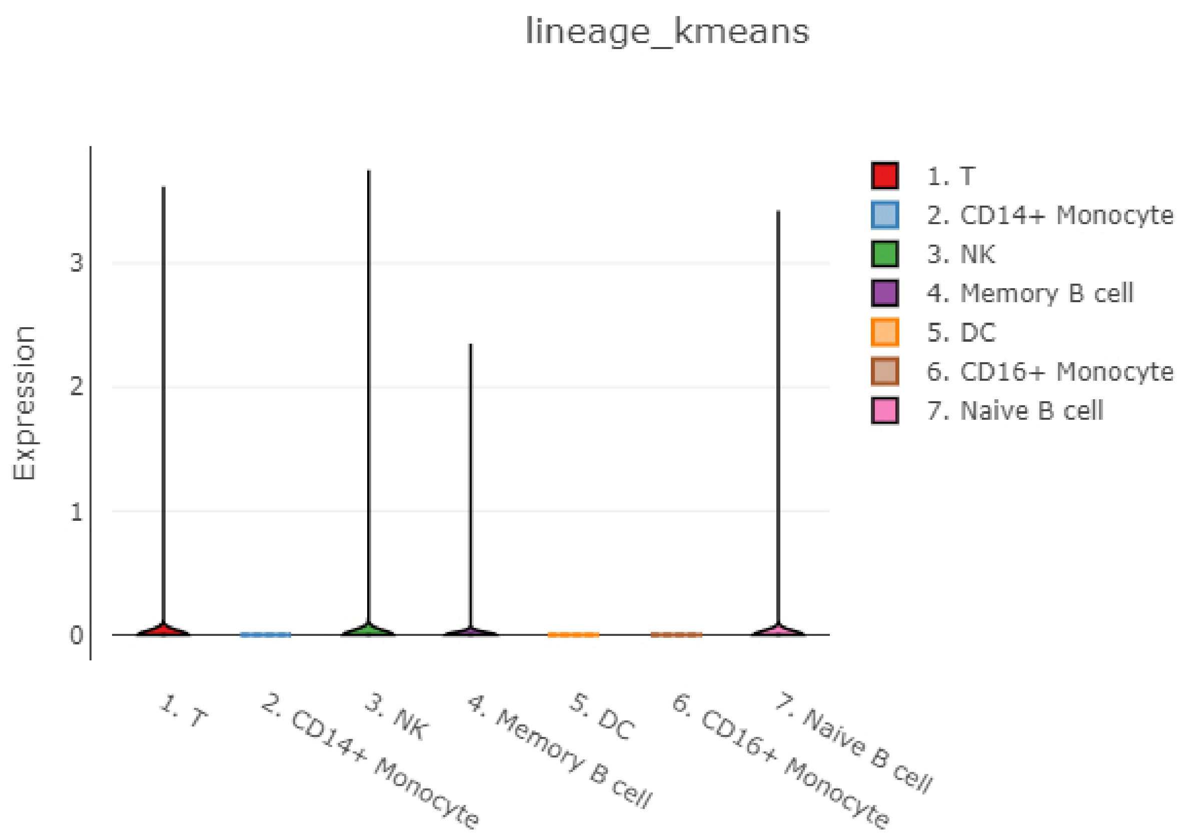

Additional File 12: Figure S3. Violin diagram of the expression of *CDC6* in the PBMCs from healthy humans.

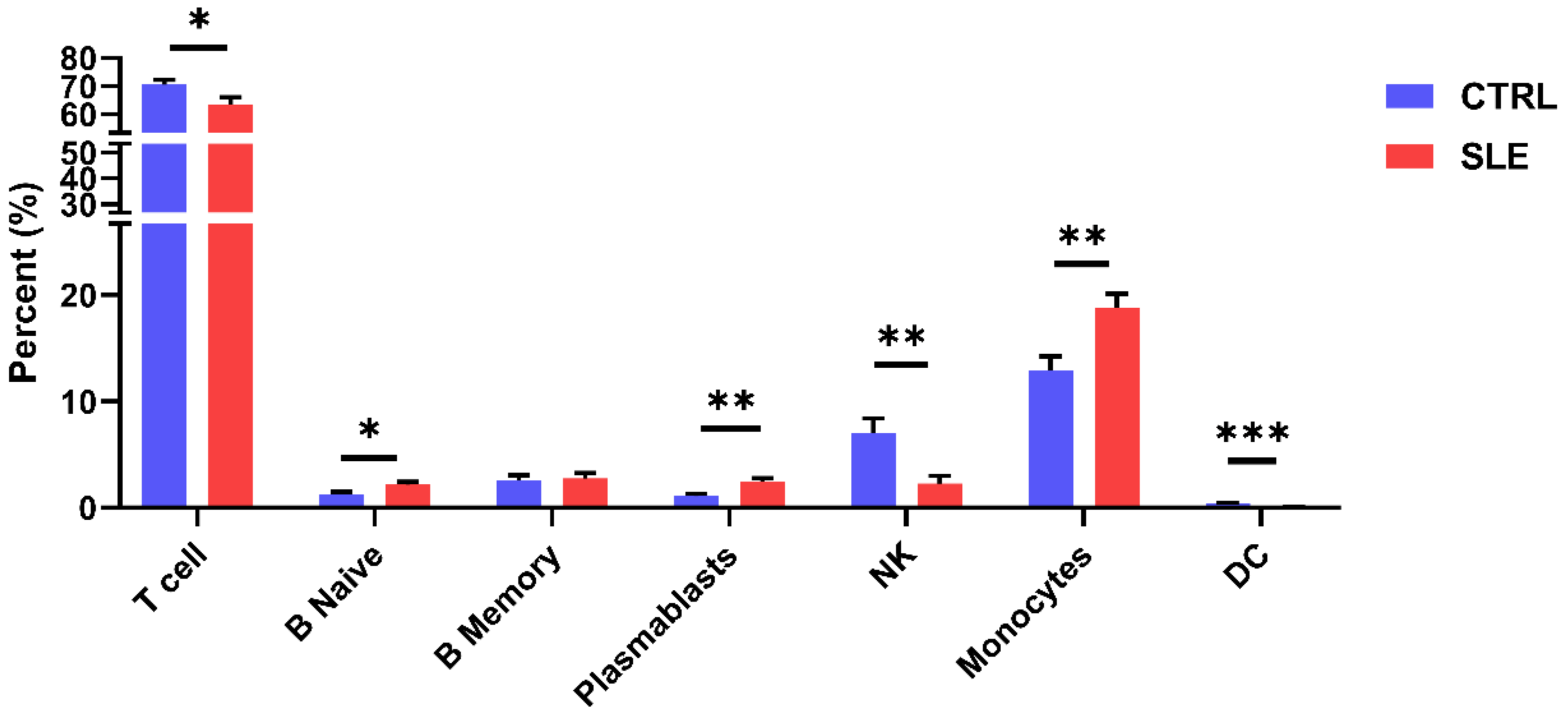

Additional File 13: Figure S4. Histogram of the proportions of various cells types in the PBMCs from SLE patients and the CTRL group. The proportions were calculated according to our sequencing data using the ABIS tool.
